# Adrenocortical Carcinoma With 2 Distinct Syndromes From Secretion of Insulin-Like Growth Factor 2 and Steroid Hormones

**DOI:** 10.1210/jcemcr/luaf078

**Published:** 2025-04-23

**Authors:** Eibhlín Marie Lonergan, Lok Yi Joyce Tan, Adrian O’Sullivan, Keizo Kanazaki, Miwa Morita, Domhnall O’Halloran

**Affiliations:** Department of Endocrinology and Diabetes, Cork University Hospital, Cork T12 DFK4, Ireland; Department of Endocrinology and Diabetes, Cork University Hospital, Cork T12 DFK4, Ireland; Department of Surgery, Cork University Hospital, Cork T12 DFK4, Ireland; Internal Medicine, Endocrinology and Metabolism, Shimane University Faculty of Medicine, Izumo 693-8501, Japan; Internal Medicine, Endocrinology and Metabolism, Shimane University Faculty of Medicine, Izumo 693-8501, Japan; Department of Endocrinology and Diabetes, Cork University Hospital, Cork T12 DFK4, Ireland; School of Medicine, University College Cork, Cork T12 AK54, Ireland

**Keywords:** adrenocortical carcinoma, insulin-like growth factor 2, androgen excess, cortisol excess, hypoglycemia

## Abstract

Non–islet cell tumor hypoglycemia as a result of insulin-like growth factor (IGF)-2 secretion is rare. A 59-year-old woman was referred with postmenopausal bleeding due to endometrial hyperplasia. Serum testosterone, estradiol, and adrenal androgens were elevated with suppressed gonadotropin concentrations. Cross-sectional imaging demonstrated a large left adrenal mass. The patient subsequently presented acutely with hypoglycemia. During a supervised fast, symptomatic hypoglycemia occurred within 5 hours. Serum samples drawn prior to hypoglycemia correction revealed an elevated IGF-2:IGF-1 ratio of 60.7 (normal <10) with low paired C-peptide and insulin, consistent with an IGF-2–secreting tumor. Hypoglycemia was managed with low-glycemic index foods and radical surgical excision was undertaken. Postoperative pathology revealed an adrenocortical carcinoma (ACC); Ki67 12%; IGF-2 positive immunostaining. This case demonstrates a rare IGF-2–secreting ACC causing clinically significant hypoglycemia with positive immunostaining for IGF-2 in addition to biochemical hyperandrogenism resulting in endometrial hyperplasia and postmenopausal bleeding. This case encompasses 2 unique syndromes from the cosecretion of both peptide factor and steroid hormone.

## Introduction

Non–islet cell tumor hypoglycemia (NICTH) as a result of insulin-like growth factor-2 (IGF-2) secretion is rare, most commonly seen in tumors of mesenchymal origin, particularly fibrous tumors, and to a lesser degree, tumors of epithelial origin [1, 2]. IGF-2 hypoglycemia (IH) associated with adrenocortical carcinoma (ACC) is less-frequently documented, with fewer than 10 cases reported [1]. IGF-2 dysregulation is implicated in tumorigenesis [3], with higher somatic expression seen in ACC when compared with benign adrenal tissues [4]. IGF-2 affinity for the insulin receptor (IR) can result in clinically significant hypoglycemia [2]. Treatment options for IH as a bridge to surgical resection of the functional tumor include frequent, low-glycemic index (GI) foods [5-8], glucocorticoids [6, 7, 9], somatostatin analogues [7, 10], growth hormone [7, 8], diazoxide [8, 9], dextrose infusion [5, 7, 8, 11, 12], and mechanistic target of rapamycin (mTOR) inhibitors [7], with varying clinical responses.

## Case Presentation

A 59-year-old woman was referred with postmenopausal bleeding secondary to endometrial hyperplasia of unknown etiology. She was otherwise well with a past medical history of atrial fibrillation and hypertension, treated with single-agent diltiazem. The patient denied any recent fluctuations in body weight.

## Diagnostic Assessment

On examination, the patient had a body mass index of 26.1 and blood pressure of 158/85 mm Hg. There were neither clinical features of Cushing syndrome nor virilization. Initial biochemical investigations showed elevated serum testosterone (144.78 ng/dL [SI: 5.02 nmol/L] [normal reference range (ref): 12.4-35.76 ng/dL (SI: 0.43-1.24 nmol/L)]), estradiol (216.81 pg/mL [SI: 796 pmol/L] [ref: 10.08-28.06 pg/mL (SI: 37-103 pmol/L)]), and adrenal androgens (androstenedione 286.4 ng/dL [SI: 10.0 nmol/L] [ref: 31.5-163.25 ng/dL (SI: 1.1-5.7 nmol/L)]; dehydroepiandrosterone (DHEA-S) 471.63 µg/dL [SI: 12.8 µmol/L] [ref: 36.85-257.92 µg/dL (SI: 1.0-7.0 µmol/L)]), with normal sex hormone–binding globulin (1069.7 µmol/dL [SI: 112.6 nmol/L] [ref: 188.1-1474.4 µmol/dL (SI: 19.8-155.2 nmol/dL)]) and androgen index 4.5 (ref: 0.5-4.7). Gonadotropin concentrations were suppressed (luteinizing hormone [LH] 2.9 IU/L [ref: 5.2-62 IU/L]; follicle-stimulating hormone [FSH] 0.2 [ref: 27.7-133.4 IU/L]). Serum 17-hydroxyprogesterone was normal (204.89 ng/dL [SI: 6.2 nmol/L] [ref: 16.52-290.80 ng/dL (SI: 0.5-8.8 nmol/L)]). Serum cortisol concentration failed to appropriately suppress with a 1-mg overnight dexamethasone suppression test (ONDST) (cortisol 5.18 µg/dL [SI: 143 nmol/L] [ref: < 1.81 µg/dL (SI: < 50 nmol/L)]). Serum potassium was low (2.8 mmol/L [ref: 3.5-5.1 mmol/L]) with a low spot urinary potassium (15 mmol/L). Urinary free cortisol was normal (40.23 µg/24 hours [SI: 111 nmol/24 hours] [ref: 0-72.46 µg/24 hours (SI: 0-200 nmol/24 hours)]). Adrenocorticotropin (ACTH) was suppressed (<3.17 pg/mL [SI: < 0.7 pmol/L] [ref: < 29.97 pg/mL (SI: < 6.6 pmol/L)]). Aldosterone:renin ratio was normal (5.62 [ref: < 24]) (aldosterone 132 pg/mL [SI: 0.37 nmol/L] [ref: 42-209 pg/mL (SI: 0.12-0.58 nmol/L)]; renin 23.5 pg/mL [SI: 0.56 pmol/L] [ref: 2.7-16.5 pg/mL (SI: 0.06-0.39 pmol/L)]). Plasma metanephrines were nonelevated (normetanephrine 388 pmol/L [ref: 0-1180 pmol/L]; metanephrine <100 pmol/L [ref: 0-510 pmol/L]; 3-methoxytyramine <100 pmol/L [ref: 0-180 pmol/L]). Glycated hemoglobin A_1c_ (HbA_1c_) was 5.6% (SI: 38 mmol/mol) (ref: < 6% [SI: < 42 mmol/mol]). Transaminases and C-reactive protein were within normal limits.

**Figure 1.**
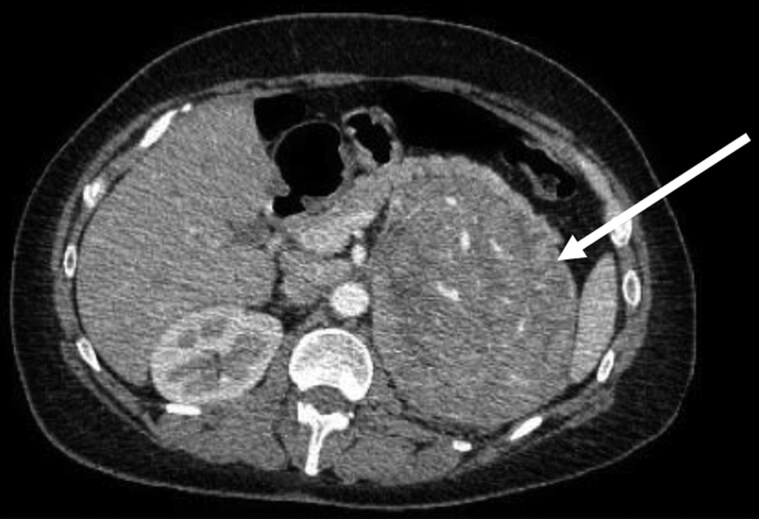
CT adrenals, axial view, showing large left-sided adrenal lesion.

Abbreviation: CT, computed tomography.

**Figure 2.**
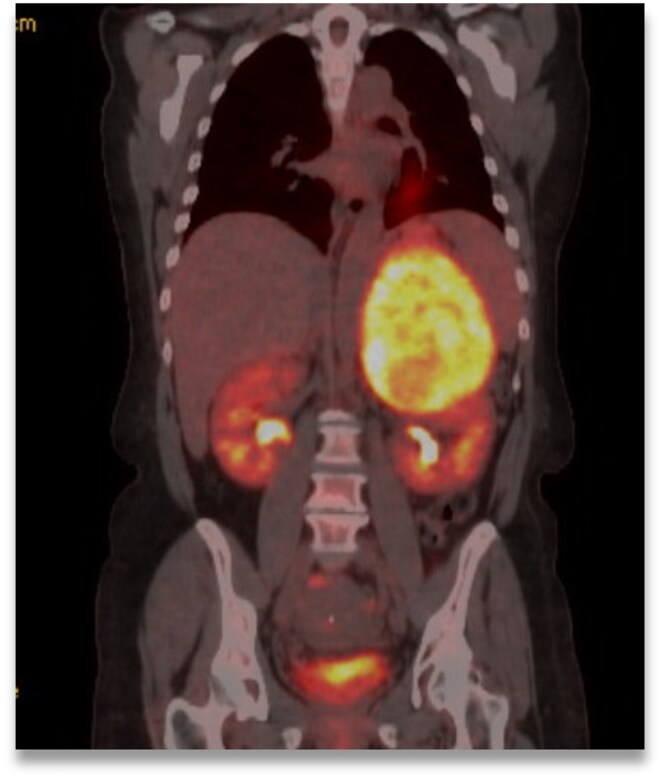
FDG-PET, coronal view, showing FDG-avid left-sided adrenal lesion.

Abbreviation: FDG-PET, fluorodeoxyglucose F18 positron emission tomography.

Computed tomography (CT) adrenals showed a 12.8- × 13.2- × 10.6-cm heterogeneous left-sided adrenal mass (Fig. 1) with evidence of intratumor necrosis and hemorrhage. The unenhanced CT attenuation was 50 Hounsfield units. The tumor compressed the upper pole of the left kidney and abutted the pancreas and spleen with renal vein invasion. Para-aortic lymph nodes were not pathologically enlarged. The mass was fluorodeoxyglucose F18 (FDG)-avid on positron emission tomography (PET) (Fig. 2) without distant metastases.

In the interim, the patient presented with an acute episode of confusion. A random point-of-care capillary blood glucose was 28.8 mg/dL (SI: 1.6 mmol/L) (ref: 70-125 mg/dL [SI: 3.9-6.9 mmol/L]). There was no history of diabetes mellitus, prolonged fasting, alcohol excess, or access to exogenous insulin or oral hypoglycemic agents. Neuroglycopenic symptoms resolved with correction of hypoglycemia. During a supervised fast, symptomatic hypoglycemia occurred within 5 hours (laboratory-confirmed serum glucose 36.0 mg/dL [SI: 2.0 mmol/L] [ref: 70-100 mg/dL (SI: 3.9-5.6 mmol/L)]). Serum samples were drawn for IGF-2, IGF-1, IGF binding protein 3 (IGFBP-3), insulin, proinsulin, and C-peptide prior to hypoglycemia correction (Table 1). Whipple triad was fulfilled [13]. Results revealed a suppressed insulin (<1.00 µIU/mL [SI: < 7 pmol/L]), C-peptide (0.06 ng/mL [SI: 16 pmol/L]), proinsulin (<2 pmol/L [SI: < 0.29 µIU/mL]), IGF-1 (13.0 ng/mL [SI: 1.7 nmol/L] [ref: 42.83-175.16 ng/mL (SI: 5.6-22.9 nmol/L)]), and IGFBP-3 (1.4 mg/dL [SI: 0.4872 nmol/L] [ref: 2.2-5.7 mg/dL (SI: 0.77-1.98 nmol/L)]), with an elevated IGF-2:IGF-1 ratio (60.7 [ref: < 10]) [2].

**Table 1.** Fasting biochemical investigations pre and post adrenal lesion excision

Investigation	Preoperative	Postoperative	Reference range
Fasting glucose	**36.0 mg/dL** **(2.0 mmol/L)**	**106.3 mg/dL** **(5.9 mmol/L)**	70-100 mg/dL (3.9-5.6 mmol/L)
IGF-2	789.37 ng/mL (103.2 nmol/L)	635.63 ng/mL (83.1 nmol/L)	ng/mL nmol/L
IGF-1	**13.0 ng/mL** **(1.7 nmol/L)**	**185.87 ng/mL** **(24.3 nmol/L)**	42.83-175.16 ng/mL (5.6-22.9 nmol/L)
IGF-2:IGF-1	**60.7**	3.4	<10
C-peptide	0.05 ng/mL (16 pmol/L)	2.50 ng/mL (828 pmol/L)	ng/mL pmol/L
Insulin	<1.00 µIU/mL (<7 pmol/L)	7.34 µIU/mL (51 pmol/L)	µIU/mL pmol/L
Testosterone	**144.78 ng/dL** **(5.02 nmol/L)**	13.56 ng/dL (0.47 nmol/L)	12.40-35.76 ng/dL (0.43-1.24 nmol/L)
Estradiol	**216.81 pg/mL** **(796 pmol/L)**	17.70 pg/mL (65 pmol/L)	10.08-28.06 pg/mL (37-103 pmol/L)
LH	**2.9 IU/L**	9.4 IU/L	5.2-62 IU/L
FSH	**0.2 IU/L**	36.5 IU/L	26.7-133.4 IU/L
Androstenedione	**286.40 ng/dL** **(10.0 nmol/L)**	**22.91 ng/dL** **(0.8 nmol/L)**	31.50-163.25 ng/dL) (1.1-5.7 nmol/L)
DHEA-S	**471.63 µg/dL** **(12.8 µmol/L)**	< **14.74 µg/dL** **(**< **0.4 µmol/L)**	36.85-257.92 µg/dL (1.0-7.0 µmol/L)
HbA_1c_	5.6% (38 mmol/mol)	**6.4%** **(47 mmol/mol)**	<6% (<42 mmol/mol)

Abnormal values are highlighted in bold font. Values in parenthesis are International System of Units (SI).

Abbreviations: DHEA-S, dehydroepiandrosterone; FSH, follicle-stimulating hormone; HbA_1c_, glycated hemoglobin A_1c_; IGF, insulin-like growth factor; LH, luteinizing hormone.

## Treatment

**Figure 3.**
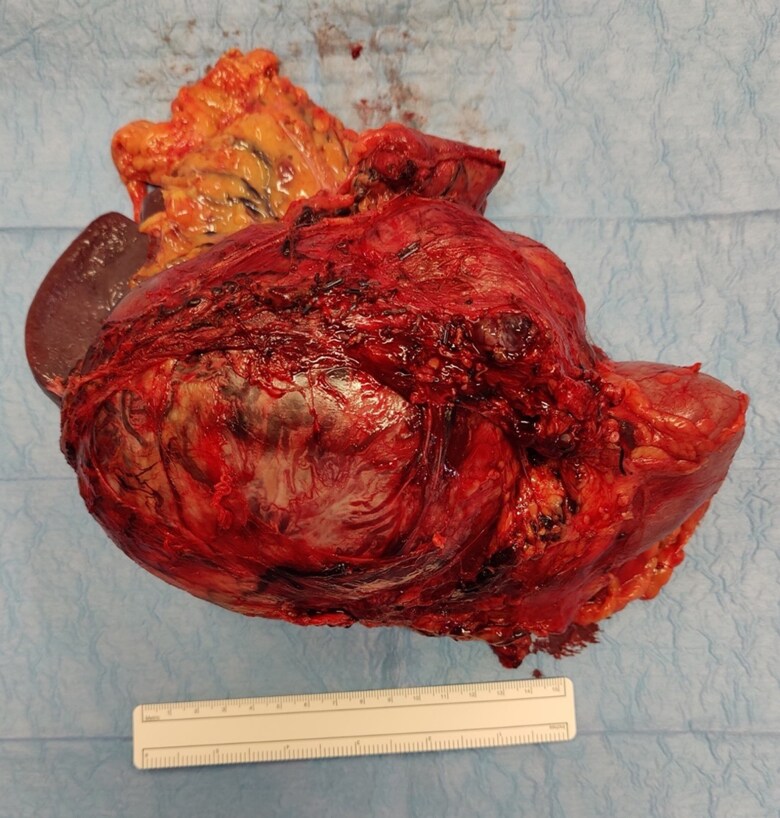
Left adrenal lesion immediately post resection.

Hypoglycemia was successfully managed with frequent, low-GI foods. The addition of glucocorticoid therapy was declined by the patient. Radical surgical excision was performed including tumor resection, left adrenalectomy, left nephrectomy, splenectomy, and distal pancreatectomy (Fig. 3). Perioperative and postoperative glucocorticoids were administered due to a previously elevated ONDST. The patient recovered without postoperative complications.

Histology revealed an ACC; 16.4 cm maximal diameter; Weiss 5 (≥3 indicating malignant behavior) [14], Aubert 6 [15] (Table 2); mitotic rate 28/50 high-power field; Ki67 12%; complete resection, negative margins (R0); and pathological staging pT2N0Mx.

**Table 2.** Weiss and Aubert tumor scoring

	Weiss	Aubert
Clear cells < 25%	1	2
Diffuse architecture > 33%	0	
Confluent necrosis	1	1
High nuclear grade	1	1
Mitotic rate > 5/50 HPF	1	2
Atypical mitoses	0	0
Venous invasion	1	
Sinusoidal invasion	0	
Capsular invasion	0	0
Total score	5	6

Abbreviation: HPF, high-power field.

**Figure 4.**
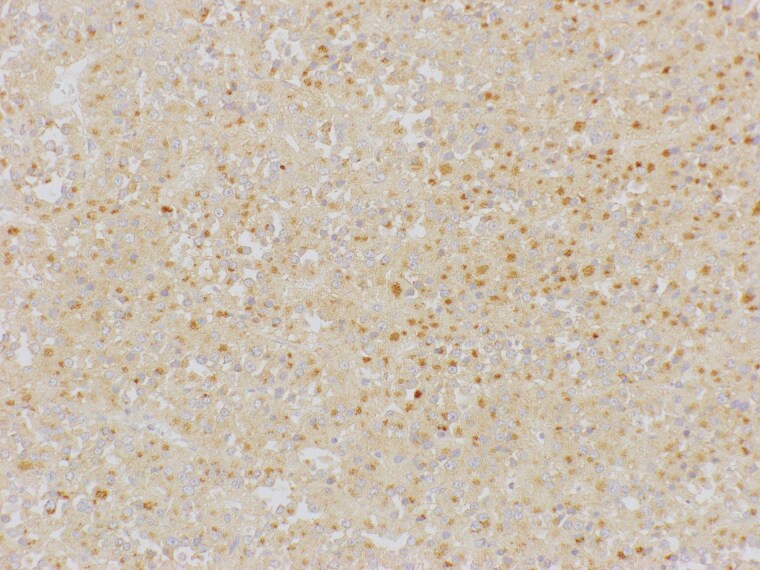
Positive insulin-like growth factor-2 (IGF-2) immunostaining of adrenocortical carcinoma (ACC), courtesy of collaboration between Cork University Hospital, Ireland, and Shimane University, Japan.

The tumor slide immunostaining was positive for IGF-2 (Fig. 4).

## Outcome and Follow-up

Postoperative biochemistry after an overnight fast revealed normalization of IGF-2:IGF-1 ratio, testosterone, estradiol, and gonadotropin concentrations, with a decrease in adrenal androgens (see Table 1). There was evidence of impaired glucose tolerance with an elevated HbA_1c_ but preserved C-peptide. Optimum glycemic control was achieved through dietary measures, without exogenous insulin requirements. The patient did not suffer from further hypoglycemia postoperatively.

Adjuvant mitotane was declined by the patient. She is currently undergoing regular radiological monitoring with CT of the thorax-abdomen-pelvis.

## Discussion

NICTH as a result of IGF-2 secretion, while uncommon, is an important presentation with considerable morbidity and mortality. The majority of cases are due to tumors of fibrous origin, followed by nonfibrous tumors of the liver [1]. There are fewer than 10 cases of IH secondary to ACC reported (Table 3).

**Table 3.** Summary of cases of insulin-like growth factor-2 secretion secondary to adrenocortical carcinoma, including demographic, biochemical, and tumor characteristics, and pharmacological treatment of hypoglycemia

Author, y	Age, y	Sex, M/F	Tumor size, cm	IGF-2 level, ng/mL; nmol/L	IGF-2:IGF-1, <10	Other hormone secretion	Metastases	Pharmacological treatment of hypoglycemia
Eguchi et al, 2001 [5]	78	F	9 × 8 × 12	889 ng/mL; 116.22 nmol/L (ref: ND)	53	Cortisol	None	ND
Korevaar et al, 2014 [7]	41	F	14 × 4.5 × 11	789.37 ng/mL; 103.2 nmol/L (ref: ND)	30.4	Androstenedione DHEA-S Testosterone	Distant	Glucocorticoids, growth hormone 8 mg/d, dextrose infusion, octreotide 100 mcg 3×/d, everolimus
Dilrukshi et al, 2020 [8]	43	M	22 × 13 × 23	832.21 ng/mL; 108.8 nmol/L (ref: ND)	38.9	ACTH Androstenedione DHEA-S	Distant	Oral dextrose, dextrose infusion, diazoxide, growth hormone 4 mg/d
Morilla et al, 2017 [9]	18	M	ND	331 ng/mL; 43.27 nmol/L (ref: 35-1000 ng/mL; 45.75-130.73 nmol/L)	13.2	ND	Distant	Dexamethasone, diazoxide 500 mg/d
Marchetti et al, 2016 [10]	21	F	15 × 15 × 14	197 ng/mL; 25.75 nmol/L (ref: 267-616 ng/mL; 34.9-80.53 nmol/L)	21.9	Cortisol DHEA-S Testosterone	Distant	Octreotide
Kim et al, 2016 [11]	39	M	18 × 16 × 9	555 ng/mL; 72.55 nmol/L (ref: 288-736 ng/mL; 37.65-96.22 nmol/L)	27.8	None	None	Dextrose infusion
Pereira et al, 2024 [12]	21	M	ND	ND	15.6	Cortisol	Distant	Dextrose infusion, glucagon infusion
Dutta et al, 2013 [16]	32	F	15.3 × 12.7 × 12	489 ng/mL; 63.92 nmol/L (ref: 288-736 ng/mL; 37.65-96.22 nmol/L)	8.6	ND	Distant	ND

Abbreviations: ACTH, adrenocorticotropin; DHEA-S, dehydroepiandrosterone; F, female; IGF, insulin-like growth factor; M, male; ND, no data; ref, reference.

IGF-2 plays a role in growth regulation, particularly in fetal development [3]. It is a peptide growth factor that acts, along with IGF-1, as the main ligand of the type 1 IGF receptor, resulting in downstream signaling and cell proliferation. IGF-2 also binds to the structurally similar IR, which is expressed in 2 isoforms, IR-A and IR-B. With an affinity for IR-A close to that of insulin, IGF-2 can exert mitogenic effects [17], but can also result in clinically significant hypoglycemia, particularly when present in circulating levels 100 to 1000 times that of insulin [2].

IGF-2 expression is differentially upregulated in ACC compared to benign adrenal lesions. One study looking at 67 samples of ACC and 64 samples of adrenal adenomas showed tissue expression of IGF-2 by immunohistochemistry (IHC) at 92.5% and 54.7%, respectively. Thus, IGF-2 plays a role as a potential additional diagnostic indicator in these tumor types [4].

A recent systematic review of IGF-2 expression in ACC and benign tissues by IHC revealed various quantitative and qualitative methods for determining IGF-2 positivity in tumor cells [18]. In our case, standard IHC analysis identified IGF-2–positive labeling of cells when compared with internal nontumor controls. Due to the unavailability of definitive positive control samples at the time of section evaluation, a quantification threshold for IGF-2 positivity was not established in the analysis. Future criteria for IGF-2 staining significance could be established by including a range of control samples to reach a threshold of positivity and associated tumor pathology with IGF-2 production.

The cosecretion of peptide and steroid hormones in this case raises the question of a possible relationship between somatic IGF-2–positive cells and steroid-producing cells. While this is not established in ACC tumors, de novo steroidogenesis has been shown to increase after treatment of androgen receptor–positive prostate cancer cells with IGF-2 via upregulated expression of steroid acute regulatory protein (StAR) as well as other steroidogenic enzymes [19]. Subsequent inhibition of the IGF-2 signaling axis attenuates these effects.

Serum hypokalemia is a feature of IGF-2–secreting tumors in up to 50% of cases. The pathophysiology is not clearly established [2]. In our case, we observed an associated low urinary potassium. This contradicts hypercortisolism as an underlying cause, a clinical scenario in which one would expect an elevated urinary potassium through mineralocorticoid receptor agonism. The high affinity of IGF-2 for IR-A could account for a transcellular shift in potassium, similar to that seen with insulin. However, this has not been investigated to date.

A systematic diagnostic approach to IH is paramount. Classic symptoms of hypoglycemia should be correlated with a low plasma glucose concentration, with resolution of symptoms once hypoglycemia is corrected, thus fulfilling Whipple triad [13]. Fasting hypoglycemia will predominate [2]. In patients without diabetes mellitus, and in the absence of exogenous insulin and oral hypoglycemic agents, acute illness, and hormone deficiencies, further investigations should be requested during a supervised fast on laboratory-confirmed, symptomatic hypoglycemia. These include insulin, C-peptide, and proinsulin initially, and IGF-2, IGF-1, and IGFBP-3 if appropriate. With IH, endogenous insulin production will be suppressed. However, serum IGF-2 concentrations are not always elevated. One study measuring serum IGF-2 and IGF-1 levels by radioimmunoassay in patients with NICTH found that only 42% of patients with IH had IGF-2 concentrations above the upper limit of normal individuals [20]. However, IGF-2:IGF-1 ratios ranged from 16.4 to 64.2, which were significantly higher than those of normal individuals. Therefore, serum IGF-2:IGF-1 ratio is a preferred biochemical marker compared to IGF-2 alone, with a ratio greater than 10 being diagnostic of IGF-2 hypersecretion [2].

Cross-sectional imaging is warranted to identify a primary tumor, with surgical resection being most effective in the definitive resolution of IH. For patients awaiting or unfit for surgery, clinical nutrition input is recommended regarding frequent, low-GI foods [5-8]. Glucocorticoids are the most common second-line option [6, 7, 9], with no consensus on preferred preparation or dosing. In the inpatient or perioperative setting, intravenous dextrose infusion is used, but is not a viable option for patients outside this setting [5, 7, 8, 11, 12]. Diazoxide was seen to be ineffective as a fourth-line option in one patient with metastatic ACC and associated IH [8]. In resistant cases, growth hormone and octreotide have both been trialed with varying efficacy [7, 8, 10]. One female patient presenting with hypoglycemia secondary to metastatic ACC was trialed on everolimus, an mTOR inhibitor previously shown to be effective in the management of insulinoma-induced hypoglycemia [21], with no clinical response [7].

IH in ACC is rare, with fewer than 10 cases reported. Higher somatic IGF-2 expression has been seen in ACC when compared with benign adrenal adenomas. Elevated circulating IGF-2 can cause clinically significant hypoglycemia through an affinity for IR-A. A stepwise approach to the diagnosis of IH is warranted through a supervised fast, with an IGF-2:IGF-1 ratio greater than 10 being diagnostic. Management options include primary tumor resection, with second-line pharmacotherapy including glucocorticoids, growth hormone, somatostatin analogues, and mTOR inhibitors, with varying clinical responses. This is a unique case of an ACC producing 2 distinct clinical syndromes due to the cosecretion of both a peptide factor and steroid hormone.

## Learning Points

- IGF-2 plays an important role in physiological cell proliferation.
- By binding the IR-A, IGF-2 can induce clinically significant hypoglycemia.
- IGF-2 expression is upregulated in ACC when compared with benign adrenal tissue and could act as an adjuvant immunohistochemical diagnostic tool.
- A systematic and comprehensive approach to the evaluation and diagnosis of IH is paramount.
- The pharmacological treatment options for IH are variable in their efficacy in the setting of ACC.

## Data Availability

Data sharing is not applicable to this article as no data sets were generated or analyzed during the present study.

## Abbreviations

ACC: adrenocortical carcinoma
ACTH: adrenocorticotropin
CT: computed tomography
DHEA-S: dehydroepiandrosterone
FDG: fluorodeoxyglucose F18
FSH: follicle-stimulating hormone
GI: glycemic index
HbA_1c_: glycated hemoglobin A_1c_
HPF: high-power field
IGF: insulin-like growth factor
IGFBP-3: IGF binding protein 3
IH: IGF-2 hypoglycemia
IHC: immunohistochemistry
IR: insulin receptor
LH: luteinizing hormone
mTOR: mechanistic target of rapamycin
NICTH: non–islet cell tumor hypoglycemia
ONDST: overnight dexamethasone suppression test
PET: positron emission tomography
ref: normal reference range
StAR: steroid acute regulatory protein
